# Refinement of Neuronal Synchronization with Gamma Oscillations in the Medial Prefrontal Cortex after Adolescence

**DOI:** 10.1371/journal.pone.0062978

**Published:** 2013-04-30

**Authors:** Julián de Almeida, Iván Jourdan, Mario Gustavo Murer, Juan E. Belforte

**Affiliations:** 1 Neural Circuit Physiology Lab, Systems Neuroscience Group, Department of Physiology and Biophysics, School of Medicine, University of Buenos Aires, Buenos Aires, Argentina; 2 Physiology of Animal Behavior Lab, Systems Neuroscience Group, Department of Physiology and Biophysics, School of Medicine, University of Buenos Aires, Buenos Aires, Argentina; 3 Instituto de Ingeniería Biomédica, School of Engineering; University of Buenos Aires, Buenos Aires, Argentina; Zhejiang University School of Medicine, China

## Abstract

The marked anatomical and functional changes taking place in the medial prefrontal cortex (PFC) during adolescence set grounds for the high incidence of neuropsychiatric disorders with adolescent onset. Although circuit refinement through synapse pruning may constitute the anatomical basis for the cognitive differences reported between adolescents and adults, a physiological correlate of circuit refinement at the level of neuronal ensembles has not been demonstrated. We have recorded neuronal activity together with local field potentials in the medial PFC of juvenile and adult mice under anesthesia, which allowed studying local functional connectivity without behavioral or sensorial interference. Entrainment of pyramidal neurons and interneurons to gamma oscillations, but not to theta or beta oscillations, was reduced after adolescence. Interneurons were synchronized to gamma oscillations across a wider area of the PFC than pyramidal neurons, and the span of interneuron synchronization was shorter in adults than juvenile mice. Thus, transition from childhood to adulthood is characterized by reduction of the strength and span of neuronal synchronization specific to gamma oscillations in the mPFC. The more restricted and weak ongoing synchronization in adults may allow a more dynamic rearrangement of neuronal ensembles during behavior and promote parallel processing of information.

## Introduction

Adolescence is a time of remarkable physical and behavioral change when individuals transition from a life of dependence on caregivers towards self-sufficiency. But it is also a period of increasing incidence of psychiatric illness. The National Comorbidity Survey Replication indicated that the peak age of onset for any mental health disorder is 14 years [1]. Anxiety disorders, bipolar disorder, depression, eating disorders, schizophrenia and substance abuse commonly emerge during adolescence [2].

During human adolescence profound structural changes take place in areas related to executive function and reward processing such as the medial prefrontal cortex (mPFC). Remarkably, structural synaptic refinement during adolescence has been demonstrated in humans [3], non-human primates [4], [5], [6], [7] and rodents [8], [9]. This “synaptic pruning” has been interpreted as a process by which ‘redundant’ synapses that were overproduced in the early years of life are eliminated resulting in a refinement of the cortical circuit [10], [11]. Interestingly, circuit refinement is especially important in areas that have coincidentally been involved in psychiatric disorders with adolescent onset [12].

Synaptic pruning is part of a more general process of brain maturation involving metabolic [13], [14], gene expression profiling [15], [16], and functional changes [17], [18], which extend the period of prefrontal development up to young adulthood in humans. Consistent with the view of protracted cortical circuit maturation, activation of frontal regions is more focused in adults than in children and adolescents during tasks involving working memory, executive control and visual processing [19], [20]. Although cortical maturation may underlie the changes that occur during adolescence in various aspects of cognitive functioning [12], [21], we still have limited information about the neurophysiological processes that link anatomical changes to cognitive functions.

During adolescence, rodents, like humans and other mammals, are faced with similar developmental challenges of acquiring the necessary skills to permit survival away from parental caretakers as they undergo puberty [22]. Like primates, rodents pass through a period of increased risk taking and novelty seeking [23], [24], [25], enhanced appetite for rewards [26], and increased anxiety [27], [28] accompanying puberty. Social behavior ontogeny in mice also resembles adolescent development of other mammalian species, with a reported peak in social play and social interaction behaviors around early adolescence -postnatal day (PD) 35- [29], accompanying the acquisition of sexual competence (for revision see [22]). Paralleling findings in primates, synaptic density declines during adolescence in rodents [8], [9], [30], [31], [32]. Moreover, the volume of the rat mPFC -assessed histologically- peaks before puberty (PD 28) and declines during adolescence towards adult levels [33]. Further supporting parallel developmental mechanisms across rodents and primates, age related gene expression trajectories are overall conserved among humans, chimpanzees, and mice [34]. The demarcation of a period resembling adolescence in rodents allowed performing detailed physiological studies that have shown a decrease in evoked postsynaptic responses in layer V pyramidal neurons of the mPFC and changes in the sensitivity of mPFC pyramidal neurons and interneurons to dopamine receptor stimulation, during adolescence [35], [36]. Though, *in vivo* studies showing a physiological maturation of the mPFC at the circuit level are lacking.

Cognitive functioning requires formation of transient coalitions of neurons, which become entrained to transient high frequency (gamma) rhythms. When a subject is not under high cognitive demand, these same neurons are entrained to resting rhythms [37], [38], [39]. Cognitive functioning may depend on the ability of neurons to disengage from ongoing rhythms to take part in a dynamic coalition during a task [40]. Although structural and functional synaptic changes occurring during adolescence may be expected to impact on neuronal synchronization, little is known about maturation of neuron entrainment to gamma rhythms in the transition from childhood to adulthood. To evaluate the physiological maturation that undergo the mPFC circuit during adolescence, we recorded pyramidal neurons and interneurons and local field potential (LFP) activity from the mPFC of juvenile and adult mice during well defined global cortical states induced by anesthesia.

## Materials and Methods

Ethics Statement: all experimental procedures were in accordance with institutional (Institutional Animal Care and Use Committee of the School of Medicine, University of Buenos Aires) and government regulations (SENASA: Servicio Nacional de Sanidad y Calidad Agroalimentaria, RS617/2002, Argentina). This study was specifically approved by the Institutional Animal Care and Use Committee of the School of Medicine, University of Buenos Aires (RS2964/2010). All efforts were made to minimize the number of animals used and their suffering. All surgeries were performed under urethane anesthesia. Fourteen male C57BL/6 mice (seven 28–32 days old and seven 3–4 month old) were maintained on a 12 h light: 12 h dark cycle, at constant temperature (21°–24°C) with free access to food and water. Adolescence in mice extends from around PD 35 to 55, and is generally considered young adults around two month of age [22].

Mice were anesthetized with urethane (1.6 g/kg, i.p.), treated with a local anesthetic in the scalp and pressure points (bupivacaine hydrochlorate solution, 5% wv/v, Durocaine, AstraZeneca S.A., Argentina 0.1–0.3 ml) and secured to a stereotaxic frame (Stoelting Co, Wood Dale, IL, USA). Temperature was maintained between 36–37°C with a servo-controlled heating pad. Additional urethane was administered as required to retain an abolished reflex response to a tail pinch [41] (customarily 0.1 to 0.2 g/kg i.p. every two to four hours).

The prevalent global brain states under urethane are characterized by either slow in the cortical EEG (“slow wave state”) or spontaneous desynchronization of the cortical EEG with theta activity in the hippocampus [42], [43] (“desynchronized state”). The cortical and hippocampal EEG (0.1–300 Hz bandwidth) were recorded through two concentric bipolar electrodes (SNE-100, Better Hospital Equipment, Rockville Centre, NY, U.S.A.) positioned at 1.8 mm anterior to bregma, 0.8 mm from midline and 1 mm from cortical surface (frontal cortex) and 2.2 mm posterior to bregma, 1.7 mm from midline and 2 mm from cortical surface (hippocampus). Custom made software allowed automatic selection of epochs with low slow wave relative power (0.45–1.8 Hz) in PFC and high theta relative power (3.5–7 Hz) in the hippocampus. At least 15 minutes of neuronal activity were analyzed for each animal.

Extracellular recordings of mPFC (1.3–2.2 mm anterior to bregma, 0.8 mm from midline with a 10° angle in the coronal plane, 1.5–2.6 mm below cortical surface) were acquired from 24 channels of a two-shank silicon probe (100 µm vertical site spacing and 500 µm horizontal shank spacing; NeuroNexus Technologies, Ann Arbor, MI). We restricted our analysis to the mPFC (infralimbic and prelimbic areas) since this region has anatomical and functional similarities with the primate dorsolateral prefrontal cortex and the anterior cingulate cortex [44], [45], [46]. Moreover, electrophysiological and computational evidence suggested that the rodent medial PFC combine elements of these two primate areas at rudimentary level [47]. Signals were amplified and band pass filtered to obtain local field potentials (LFP; 4–300 Hz) and multiunitary action potential activity (300–3000 Hz) stemming from the same recording sites. All signals were digitized at 25 kHz and stored in a computer for offline analysis. Spike sorting was performed off line as described in [48] by using *wave_clus* [49]. Well isolated units were classified off-line according to published criteria as interneurons or putative pyramidal neurons based on spike waveforms [50].

Custom made Matlab routines were used to determine the relationship between spike discharges and frequency components of the LFP in the desynchronized state. The LFP was digitally filtered (zero phase-lag Butterworth digital filter) to obtain the following band-passed waveforms: 4–7 (theta), 8–12 (alpha), 13–30 (beta), 30–48 (low gamma) and 52–80 Hz (high gamma). A Hilbert transform was used to obtain the phase angle at every point of each waveform (“instantaneous phase”). Next, the number of spikes occurring at different phase angles of each waveform was depicted in circular plots (bin size: 10°). Phase locking of spike discharges to a given frequency band was determined by assessing deviation from uniformity in these circular plots with the Rayleigh test. The strength of synchronization was further assessed by comparing the module of the resultant vectors summarizing these circular distributions [51]. Units with less than 100 recorded spikes were excluded from this analysis. Statistical analysis (including student t-test, two way ANOVAs followed by Newman-Keuls post hoc test, Fisher exact test for proportions) was performed with Statistica 7 (StatSoft Inc., OK, USA) and fitting curves were obtained with Prism 4.0 (Graphpad Software, CA, USA).

At the end of each experiment, animals received a lethal dose of urethane and were transcardially perfused with 10 ml cold saline solution followed by 20 ml of buffered paraformaldehyde (4% w/v in 0.1 M phosphate-buffer, PB). Brains were removed, immersed for 30–45 minutes in the same fixative at room temperature, and stored in 0.1 M PB containing 15% sucrose at 4°C for 24–72 hours. Coronal brain sections were cut with a freezing microtome (50 µm) for histological reconstructions. Location of the concentric bipolar electrodes was assessed by visual examination of the mechanical tissue damage in the coronal sections using a transmitted light microscope at low magnification. In order to determine the location of the silicon probe recording sites, before each experiment the multi-electrode was immersed in a red fluorescent dye (1,1′-dioctadecyl-3,3,3′,3′-tetramethylindocarbocyanine perchlorate, 100 mg/ml in acetone; DiI, Molecular Probes) and air dried for 30 minutes before use. This allowed detecting the fluorescent material deposited in the tissue with an epifluorescence microscope. In all cases, photomicrographies were obtained from sections of interest for subsequent reconstruction of recording sites.

## Results

### Global Brain States are Comparable in Juvenile and Adult Mice

**Figure 1:**
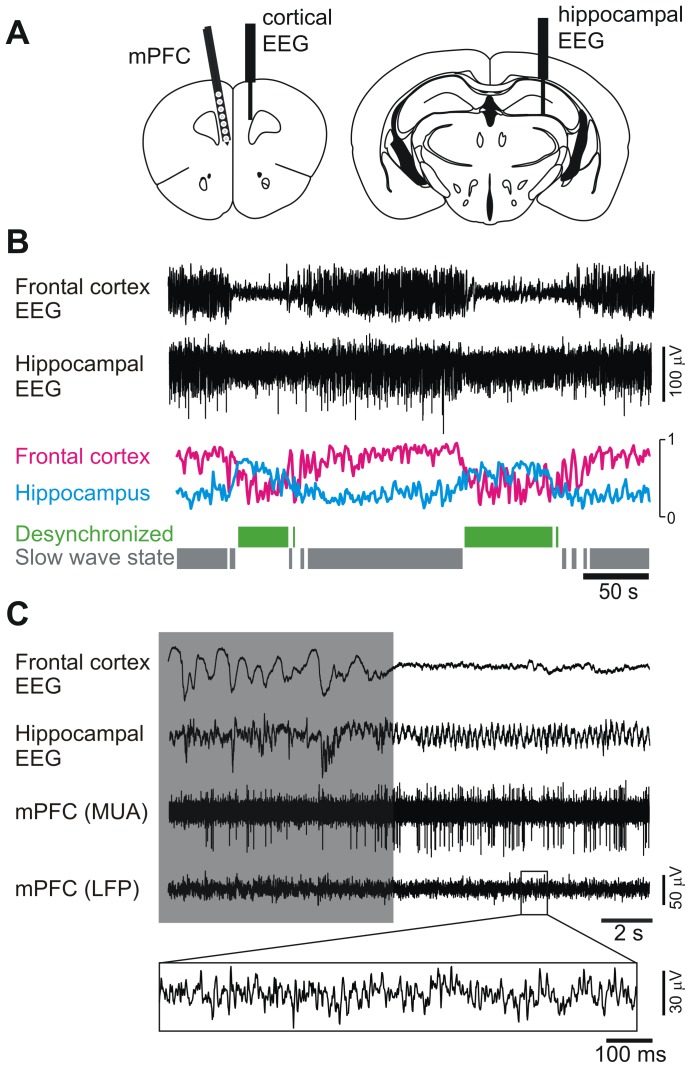
Recording of mPFC activity under different global brain states. **A.** Positioning of a multichannel recording “silicon probe” in the medial PFC (left) and of bipolar electrodes in the frontal cortex (cortical EEG) and hippocampus (hippocampal EEG, right). **B.** Representative example showing the cortical and hippocampal EEG patterns seen in mice under urethane anesthesia, the mirror changes in relative power taking place in a low frequency band recorded from the frontal cortex (purple trace) and the theta band recorded from the hippocampus (light blue), and the epochs selected automatically by the algorithm used to classify the global brain states (green and grey). **C.** Representative recording during a transition from the slow wave state (box) to the desynchronized state. Note at right the lower amplitude and higher frequency of the cortical EEG accompanied with theta activity in the hippocampus, which are characteristic of the desynchronized state. The two lower traces show activity in one channel of the silicon probe after being bandpass filtered to separate multiunit activity (MUA) and the local field potential (LFP).

**Figure 2:**
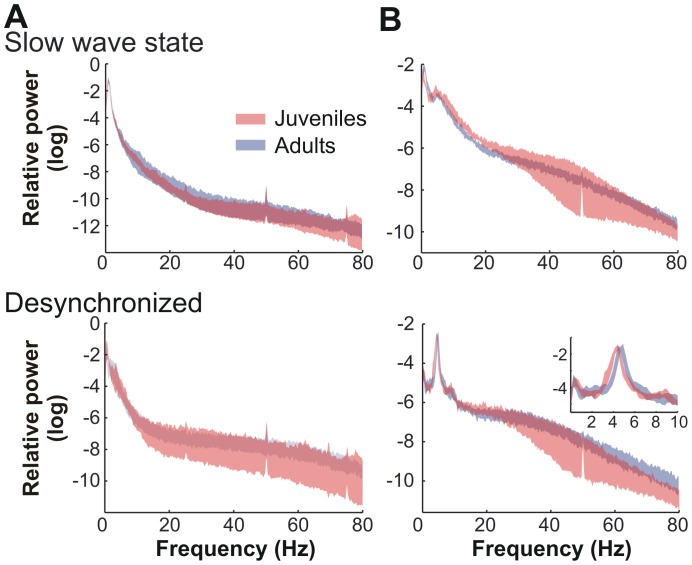
Juvenile and adult mice show similar global brain states under urethane anesthesia. Relative power spectra corresponding to cortical (**A**) and hippocampal EEG (**B**), in juvenile and adult mice, in slow wave and desynchronized states. The data are the relative power encompassed between the 95% confidence intervals of the mean. Juveniles and adults show similar frequency composition of cortical and hippocampal EEG, but hippocampal theta shows a slightly higher frequency in adults (4.33±0.07 vs. 4.67±0.08 Hz; p<0.01, t-test).

To analyze the functional changes induced by adolescent maturation in the mPFC we measured local field potentials (LFPs) together with neuronal firing activity in juvenile and adult wild type mice with a multichannel electrode (Figure 1A). The use of general anesthesia to explore mPFC properties allow us to minimize the impact of peripheral inputs that may vary across developmental stages [52], [53]. Mice anesthetized with urethane display well characterized global brain states (Figure 1B–C). The prevalent slow wave state shows coordinated delta oscillatory activity across the cortex resembling natural Slow Wave Sleep. However, cortical activity spontaneously switches back and forth to a desynchronized state with enhanced high-frequency components resembling wakefulness EEG [54]. Frontal cortex electrocorticogram (cortical EEG) and hippocampal global activity (hippocampal EEG) were simultaneously recorded with macroelectrodes to define epochs of slow wave and desynchronized states (Figure 1B–C). To avoid any sampling bias in state identification a custom made algorithm was used to automatically identify periods of each brain state from the entire recording session (Figure 1B). Fourier analysis of frequency components for each global brain state showed no significant differences in frontal cortex activity between epochs extracted from juvenile and adult mice (Figure 2A). A small but significant shift was observed for peak frequency of hippocampal theta oscillation between juveniles and adults (Figure 2B, inset). Similar results were obtained for the analysis of LFPs recorded from the mPFC with the multichannel electrode, during the slow wave and desynchronized states (Figure S1 in File S1), suggesting that global cortical rhythms under anesthesia are not markedly affected by adolescent maturation.

### mPFC Neurons are Differentially Entrained by Local Rhythms

We next focused our attention to neural activity from mPFC neurons. Due to similarities in network dynamics of the desynchronized state seen under urethane with the awaken state we restricted the analysis of neuronal activity to this condition [54], [55], [56]. We recorded a total of 26 interneurons from 102 recording sites in 7 juvenile mice and 39 interneurons from 101 recording sites in 7 adult wild-type mice. Units were classified off-line according to published criteria as interneurons or pyramidal neurons based on spike waveforms (Figure S2 in File S1). After identifying a putative interneuron, we isolated pyramidal neurons (32 in juvenile and 35 in adult mice) from the same or adjacent recording sites for comparative purposes. Seen through a multichannel electrode, located at a pre-established stereotaxic position along the mPFC, the total number of spikes per second measured for both cell types was not affected by adolescent maturation (pyramidal neurons: 2.81±0.41 in juveniles vs. 3.06±0.40 in adults, p = 0.66, t-test; interneurons: 3.49±0.55 in juveniles vs. 4.79±1.02 in adults, p = 0.33, t-test). However, the temporospatial organization of spike firing in the mPFC differed between adolescents and adults.

**Figure 3:**
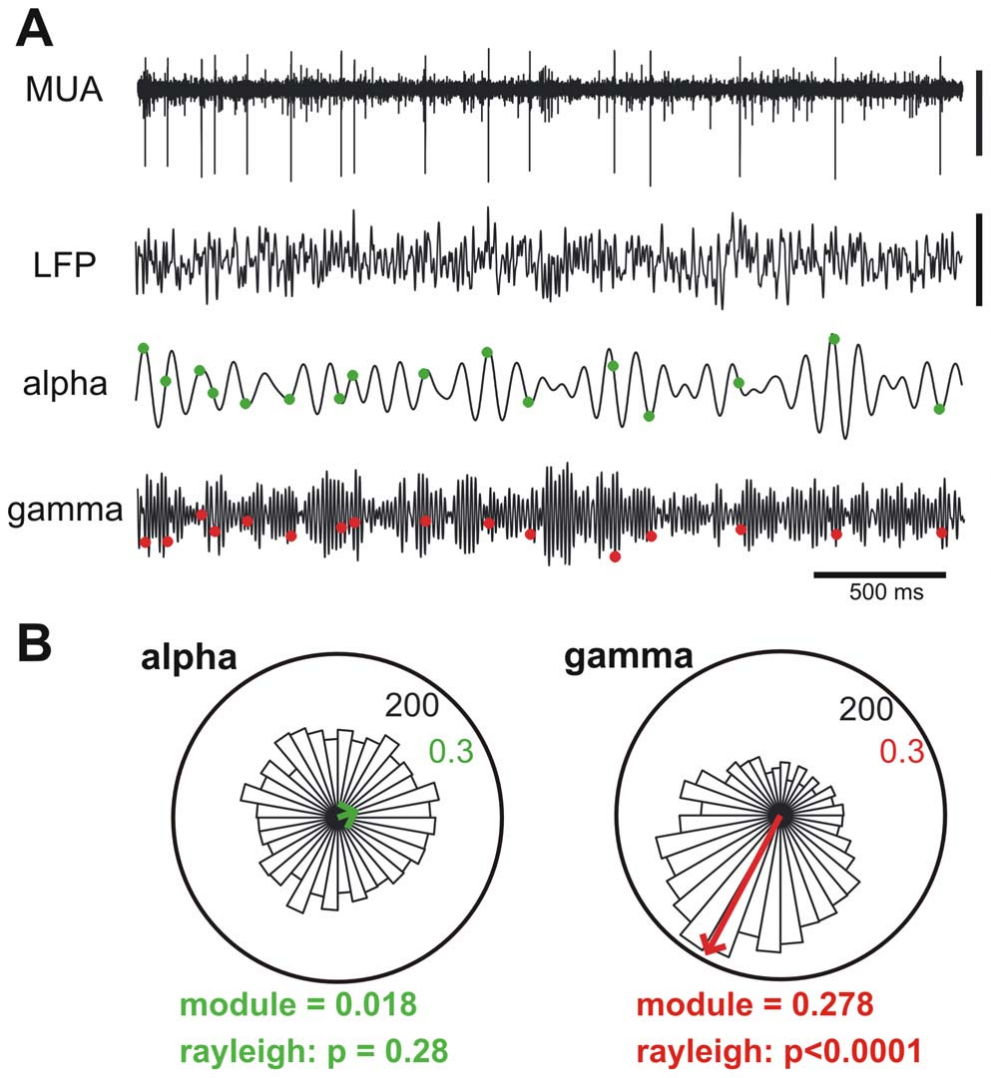
Neurons synchronize preferentially to gamma activity in the mPFC. **A.** Representative traces showing the raw multiunit activity (MUA) and local field potential (LFP) recorded through one contact of a silicon probe located in the mPFC of an adolescent mouse. The traces shown below show signals resulting from band-pass digital filtering the LFP for alpha and high gamma bands. The green and red dots mark the occurrence of spikes from a pyramidal neuron sorted from MUA. Note the preferential occurrence of spikes in the troughs of the gamma oscillation. **B.** Spike-phase plots (bin size 10°) showing a uniform distribution of spikes across phases for the alpha band and a markedly non-uniform distribution for the same spikes across phases for the gamma band (statistically evaluated by the Rayleigh test). The plots correspond to the neuron shown in A. The resultant vector of the distribution has an angle (locking phase) and a length or module (locking strength). The numbers at the upper right quadrant of the plots refer to the radial axes of frequency distribution and vector length.

Neural networks do not only encode information by unitary firing rate but also by the relative timing of spikes in the neuronal population with respect to an ongoing brain oscillation [37], [38], [39]. Entrainment of neural firing to oscillations is not only seen during cognitive activity, but also during quiet resting [57], sleep [58], and anesthesia [59]. Under these resting conditions, entrainment of firing to ongoing oscillations tells about the network connectivity [60]. To explore the impact of adolescent maturation in neural synchronization we measured the entrainment of all active sites in mPFC (including multi-unit and single-unit recordings) to different LFP oscillations in juvenile and adult mice. For any neuron or recording site, synchronization was assessed by plotting the rate of occurrence of spikes as function of the phase of an oscillation, where oscillations were all the physiologically relevant bands in the LFP, covering the spectrum from low (theta: 4 to 7 Hz) to high frequency oscillations (high-gamma 50 to 80 Hz). Thus, if a neuron fires preferentially at the trough of a given oscillation, this will produce an asymmetric circular distribution with a peak at 180°. This asymmetry can be summarized with a vector whose angle and length are the phase and strength of entrainment, respectively (Figure 3) Units were considered entrained to a given band when exhibited a statistically significant asymmetrical distribution (p<0.05 Rayleigh test). Statistical analysis revealed a complex pattern of neuronal coupling, with only a few units displaying entrainment to all frequency bands (pyramidal 1.5%, interneurons 3.1%) and others entrained to specific bands only (Figure 3B). Regardless of age, the proportion of entrained units, as well as the strength of coupling, increased from low to high frequencies, with only a small fraction of units with significant entrainment in theta and alpha bands for both cell types (pyramidal: 17.9% in theta, 14.9% in alpha; interneurons: 24.6% in theta, 20.0% in alpha). In contrast, more than 70% of the pyramidal cells and interneurons were entrained to high gamma. We found that there is no contribution of spike waveform contamination to LFP locking and, in our conditions, this factor cannot account for the results we have observed (Figure S3 in File S1). This complex pattern of oscillatory entrainment opens the possibility that developmental changes during adolescence differentially affect channels of information processing linked to specific oscillatory rhythms.

### Adolescent Maturation Results in Refined Entrainment of mPFC Neurons to Gamma Oscillations

**Figure 4:**
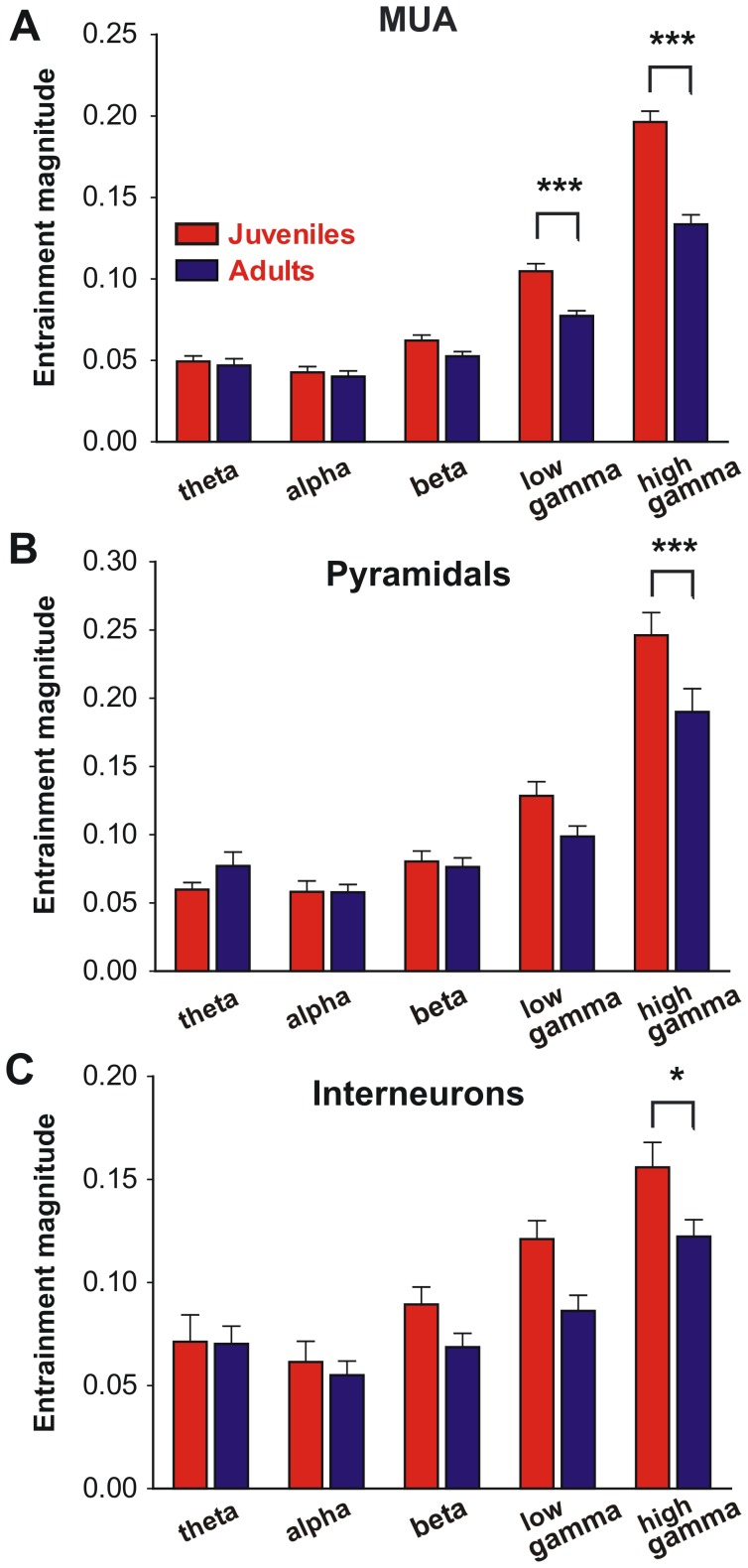
Selective reduction of gamma entrainment of mPFC neurons during adolescence. The strength of synchronization between spikes and LFP in the mPFC was assessed by computing the resultant vector of circular distributions as shown in Figure 3. Entrainment magnitude refers to the average vector length ± SEM across units. In **A**, all spikes in each mPFC recording site (95 sites in juveniles, 98 sites in adults) were used to compute the circular distributions (multiunit activity). **B** shows the average vector length for all well sorted pyramidal neurons (32 in juvenile and 35 in adult mice), and **C** shows average vector length for all well isolated interneurons (25 in juvenile and 38 in adult mice). Statistical analysis showed significant interaction between age and oscillatory band in all cases, repeated measures ANOVA interaction: MUA F_4,764_ = 21.0 p<0.0001, pyramidal cells F_4,260_ = 4.37 p<0.005, interneurons F_4,244_ = 2.47 p<0.05; *p<0.05; ***p<0.001 juvenile vs. adult Newman-Keuls post hoc test.

Adolescence has been associated with changes in circuit wiring, as seen at the level of synaptic spines [61] and brain imaging [62], which could have a parallel in neural synchrony. Analysis of juvenile versus adult mice showed that adolescent maturation results in a significant reduction of entrainment of mPFC neurons restricted to gamma oscillations (Figure 4 and Figure S4 in File S1). Reduction in strength of gamma entrainment in adults was accompanied by a global decrease in number of recording sites significantly entrained to high-gamma oscillations (MUA: 94.7% in juvenile 80.6% in adults p<0.005, Fisher exact test). No significant differences were observed in entrainment of neurons to theta and alpha oscillations between juveniles and adults, neither in multiunit recordings (Figure 4A) nor in well isolated pyramidal cells (Figure 4B) or interneurons (Figure 4C). While an important number of recording sites showed significant coupling in the beta band (pyramidal: 40.6% juveniles and 31.4% adults; interneurons: 46.1% juveniles and 33.3% adults) no significant differences were obtained in beta entrainment of pyramidal neurons and interneurons between juvenile and adults (Figure 4A–C), reinforcing the notion of a selective reduction in the gamma range. The reduction in entrainment strength to gamma is robust and can be demonstrated in multiunit recordings (Figure 4A), across the whole population of well isolated pyramidal cells and interneurons (Figure 4B–C), or when considering exclusively those well isolated neurons showing significantly asymmetric circular distributions (data no shown). Interestingly, comparison of high-gamma entrainment show significantly higher values for pyramidal cells than for interneurons irrespectively of age (pyramidal cells: 0.22±0.01 vs. interneurons: 0.14±0.07, p<0.0001, t-test, juveniles and adults pooled together) although the entrainment angle was similar regardless of age and neuron type (data not shown). To exclude the possibility that the entrainment reduction observed between ages could emerge as a consequence of a differential impact of anesthesia we calculated an anesthesia-depth index for the whole recording session for each animal (Figure S5 in File S1). No significant correlation was detected between the anesthesia level and neuronal high-gamma entrainment, neither in juvenile nor in adults (Figure S5 in File S1). Also, no significant differences were observed in the anesthesia index between juvenile and adults (2.18±0.12 vs. 2.33±0.15 respectively, student test t12 = 0.73 p = 0.47). Moreover, the same age-related differences were obtained for high-gamma entrainment in a complete different anesthetic state (Figure S6 in File S1) ruling out a possible bias of entrainment measurement due to anesthesia differences between juvenile and adults.

These results support the view that transition from pre-adolescent stage to adulthood is accompanied by a shift from generalized unit entrainment to gamma oscillations towards a more finely tuned coupling, without changes in the overall level of activity in mPFC. This refinement occurs in parallel for pyramidal cells and interneurons, suggesting that a pre-established balance between excitation and inhibition is maintained into adulthood.

### Increased Spatial Resolution for Interneuron Entrainment to Gamma Oscillations after Adolescence

**Figure 5:**
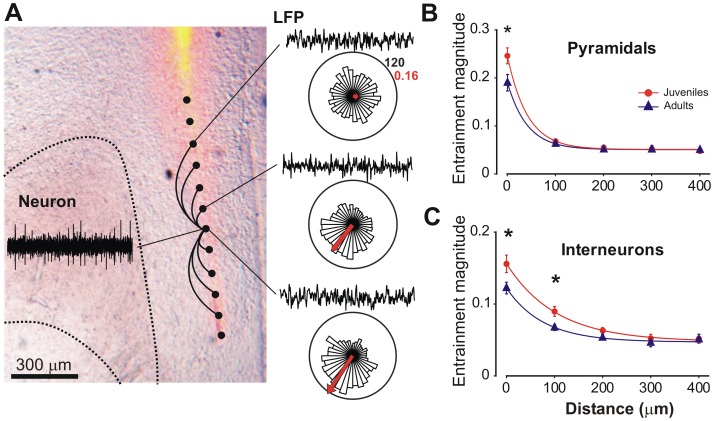
Reduced span of interneuron entrainment to gamma oscillations in adults. **A.** Schematic representation showing the localization of recording sites in the mPFC. A coronal section of a representative animal was photographed under transmitted light and epifluorescence to show the location of fluorescent material left by the labeled silicon probe in the mPFC. To estimate the span of neuronal synchronization to gamma oscillations, we took the spikes of a given recording site and computed their spike-phase circular distribution for the LFP in the recording site from which they were recorded and for LFPs at other recording sites along the electrode. In this example, three circular distributions are depicted for one unit, representing the entrainment with the local LFP (cero distance) and LFPs obtained 100 and 400 µm apart. **B** and **C.** Entrainment strength to high-gamma oscillations as function of distance along the mPFC, for pyramidal neurons (**B**) and interneurons (**C**). In adults, interneuron entrainment to high gamma is more spatially restricted than in juveniles. * p<0.05 Newman-Keuls post hoc test after a two way ANOVA for repeated measures with significant interaction. Note that pyramidal neurons show a very local entrainment to gamma, whereas interneurons are coupled to gamma recorded 200 µm apart. Entrainment magnitude refers to the average vector length ± SEM at each LFP recording site along the mPFC.

Self-organized gamma oscillations in the neocortex are transient, highly localized and emerge simultaneously at multiple cortical locations [63]. To investigate if the refinement we observed after adolescence affects the spatial organization of the mPFC we evaluated the decay with distance of gamma entrainment in juvenile and adult mice. Due to the regular geometry of the multichannel electrode used in our recordings we have been able to calculate the entrainment of a particular neuron to LFPs obtained from the neuron’s recording site and from regularly-spaced distant ones (Figure 5A).

A global analysis of our results shows that entrainment of pyramidal units and interneurons decays exponentially with distance in both, juvenile and adult groups (Figure 5B, C). However, entrainment decays more abruptly for pyramidal cells than interneurons, independently of the animals age (space constant for interneurons 92.6±11.5 µm, pyramidal 41.6±8.6 µm; p<0.0001 extra-sum-of-squares F-test, F_2,649_ = 57.7). When developmental stage is considered, we found that adolescence maturation does not reduce the entrainment of pyramidal cells to high-gamma oscillations recorded from sites 100 microns away or farther (Figure 5B). In contrast, the span of interneuron entrainment is significantly affected by adolescence maturation, with a significant reduction of entrainment to LFPs recorded 100 microns apart, suggesting that gamma entrainment is more spatially circumscribed in adults than in juveniles (Figure 5C). Interestingly, this reduction does not completely eliminate the coupling between sites since further reduction occurs at longer distances even in adults (entrainment at 100 µm vs. 400 µm adults p<0.05). Entrainment reaches a minimum value when distance is 300 µm for both cell types, confirming the local nature of gamma oscillations. Thus, the data show a more spatially compact functional network in the adult, with interneurons entrainment to gamma oscillations refined and reduced.

## Discussion

Here we show for the first time that adolescent maturation results in a reduction of the strength and span of neuronal synchronization specific to gamma oscillations in the medial prefrontal cortex. This functional refinement occurs in parallel in pyramidal cells and interneurons without affecting either the level of neuronal activity or the global brain oscillatory rhythms. Interestingly, pyramidal cells exhibit stronger synchronization with local-gamma rhythm than interneurons; however their spatial synchronization is more circumscribed supporting the notion that interneurons integrate information over wider areas than pyramidal cells [60]. Notably, the spatial synchronization of interneurons, but not pyramidal cells, is reduced after adolescent maturation, as reflected by the reduction in magnitude of gamma-entrainment to distant sites within the mPFC. The 100 µm span between recording sites in the electrode arrays may have limited our analysis of spatial entrainment in the PFC. This is specially true for pyramidal neuron recordings, which showed no clear entrainment to the LFP recorded 100 µm away in the array. Of note, the same recording array was sufficient to reveal a reduction in interneuron spatial entrainment in adults. Whether a similar developmental effect occurs in pyramidal neurons will require the use of probes with higher density of contacts, to extend our measurements below the 100 µm limit.

Proper mPFC function depends on maintaining the correct excitation-inhibition balance. As describe above, synaptic pruning of excitatory inputs occurs during adolescence, concomitantly with a remodeling of the GABAergic local system. For instance, in nonhuman primates, the density of GABA interneuron processes increases during the prepubertal period, peaks during adolescence and then sharply declines [7], [64], with similar changes also reported in rodents. Thus, evidence shows that both components of the excitation-inhibition balance are remodeled in parallel during this period. We are here providing evidence that these anatomical and histochemical changes are paralleled by a functional refinement in neuronal entrainment to cortical rhythms. Interestingly, these changes are asymmetrical, affecting the spatial synchrony with gamma oscillations particularly in interneurons.

Based on our results we propose that increased segregation of functional modules of pyramidal cells, by their relative coupling to gamma rhythms during adolescence, results in a prefrontal cortex with greater capacity of parallel processing. In this way, the adult network will be spatially more compact, with functional modules of pyramidal cells fully consolidated and resources previously recruited in basal computations now available to participate in high demanding cognitive tasks. Consistent with this view our results show that vast majority of pyramidal cells of juvenile mice are highly entrained to gamma oscillations in resting conditions with reduced sensory-motivational inputs. Transition into adulthood results in a shift from generalized neuron entrainment to gamma oscillations towards a more finely tuned coupling, with fewer units significantly entrained and reduced magnitude of coupling per cell.

Gamma oscillations are thought to provide a temporal structure for information processing in the brain [65] and have received great attention given their putative role in cognition [66], [67]. In addition, abnormal gamma oscillation and synchrony have been extensively reported in relation to psychiatric disorders, including autism [68], and particularly in schizophrenia [69], [70]. Moreover, abnormal gamma rhythms described in schizophrenia patients have been postulated as a determinant factor in the pathophysiology of the disorder [71], [72], [73], and even proposed as endophenotype for schizophrenia [74]; however, in spite of the abundant literature, a clear picture has not yet emerged. An important number of publications have reported decreased gamma oscillations in schizophrenia patients during several cognitive tasks [70], [75], [76], [77], nevertheless, inconsistent and opposite findings exist [78], [79]. Since cortical gamma oscillations are controlled by fast-spiking interneurons [80], it has been proposed that abnormal maturation of this cell population may underlie many of the schizophrenia symptoms [73]. Indeed, accumulating evidence has connected interneurons with schizophrenia pathology (reviewed in [81], [82]). Based on our results we speculated that, an abnormal maturation of the refinement we described for neuronal synchronization with gamma oscillations, could be involved in the pathophysiology of schizophrenia and other disorders with a clear developmental component during adolescence. In this way, impaired adolescent refinement will render cortical network in a gamma-over-synchronized state, with excessive neuronal resources engaged in gamma oscillations during basal demands. This excessive allocation of resources will produce a “noisy” baseline with few neurons left available to be recruited by gamma rhythms during cognitive tasks. Accordingly, several independent groups have reported a decrease in evoked-gamma oscillations and gamma synchrony in schizophrenia patients [77], [83], [84]. Notably, measurement of resting-state activity has shown higher levels of gamma oscillations in non-medicated schizophrenia patients [85], supporting the idea of a reduced dynamic range for gamma entrainment as consequence of a higher baseline.

Differences in global neural activity and connectivity in adolescent prefrontal cortex, compared with adults, have been previously described in humans [86], [87], [88], however, little is known about the precise nature of these age-related disparities at the neuronal level. Our results show that maturation during adolescence can result in changes in neuronal synchronization without alterations in the magnitude of gamma oscillation or mean firing rate. Since analysis of gamma oscillations in human studies are usually restricted to EEG-LFP signals without access to neuronal activity, abnormalities involved in psychiatric disorders may be difficult to assess with the usual diagnostic techniques.

Core physiological properties of the desynchronized EEG, like persistent up states sustained by balanced excitation and inhibition, are very similar in the awake and anesthetized preparation [55], reviewed by [56]. Fast rhythms –including gamma oscillations– appear in the cortex during brain-active states in awake, REM sleep, and in desynchronized state under anesthesia, probably engaging similar, although not identical, neuronal networks and dynamics [54]. Here, we have exploited the self-activation capacity of the anesthetized cortex to show how pyramidal cells and interneurons are recruited during spontaneous network activity in conditions of controlled global brain state and sensorimotor demands. Neuronal entrainment in awake animals may be under the influence of age related changes in sleep/wake parameters [89], sensorimotor activity, anxiety levels and other factors which could partially deviate neuronal entrainment from the developmental trajectory that we describe in this work. Further studies will be needed to understand the interaction between internal dynamics of the mPFC and external environments during development.

In summary, our results suggest that adolescent maturation results in a refinement of mPFC neurons engagement in oscillatory rhythms commonly associated with cognitive performance together with an increased spatial segregation of processing channels.

## Supporting Information

File S1 **This file contains figures relating to: Figure S1.** Relative power spectra corresponding to LFPs recorded from the mPFC. **Figure S2.** Separation of putative cortical interneurons and pyramidal cells. **Figure S3.** Phase-locking analysis results for real and simulated cases. **Figure S4.** Representative traces. **Figure S5.** No significant correlation between anesthesia level and high-gamma entrainment in juvenile or adult mice. **Figure S6.** Lack of effect of anesthesia level on age dependent high-gamma neuronal entrainment.
(PDF)

## References

[1] Kessler RC, Berglund P, Demler O, Jin R, Merikangas KR, et al (2005) Lifetime prevalence and age-of-onset distributions of DSM-IV disorders in the National Comorbidity Survey Replication. Arch Gen Psychiatry 62: 593–602. 15939837 10.1001/archpsyc.62.6.593

[2] Paus T, Keshavan M, Giedd JN (2008) Why do many psychiatric disorders emerge during adolescence? Nat Rev Neurosci 9: 947–957. 19002191 10.1038/nrn2513 PMC2762785

[3] Huttenlocher PR (1979) Synaptic density in human frontal cortex - developmental changes and effects of aging. Brain Res 163: 195–205. 427544 10.1016/0006-8993(79)90349-4

[4] Rakic P, Bourgeois JP, Eckenhoff MF, Zecevic N, Goldman-Rakic PS (1986) Concurrent overproduction of synapses in diverse regions of the primate cerebral cortex. Science 232: 232–235. 3952506 10.1126/science.3952506

[5] Zecevic N, Rakic P (1991) Synaptogenesis in monkey somatosensory cortex. Cereb Cortex 1: 510–23. 1822755 10.1093/cercor/1.6.510

[6] Bourgeois JP, Goldman-Rakic PS, Rakic P (1994) Synaptogenesis in the prefrontal cortex of rhesus monkeys. Cereb Cortex 4: 78–96. 8180493 10.1093/cercor/4.1.78

[7] Anderson SA, Classey JD, Conde F, Lund JS, Lewis DA (1995) Synchronous development of pyramidal neuron dendritic spines and parvalbumin-immunoreactive chandelier neuron axon terminals in layer III of monkey prefrontal cortex. Neuroscience 67: 7–22. 7477911 10.1016/0306-4522(95)00051-j

[8] Zuo Y, Lin A, Chang P, Gan WB (2005) Development of long-term dendritic spine stability in diverse regions of cerebral cortex. Neuron 46: 181–189. 15848798 10.1016/j.neuron.2005.04.001

[9] Zuo Y, Yang G, Kwon E, Gan WB (2005) Long-term sensory deprivation prevents dendritic spine loss in primary somatosensory cortex. Nature 436: 261–265. 16015331 10.1038/nature03715

[10] Changeux JP, Danchin A (1976) Selective stabilisation of developing synapses as a mechanism for the specification of neuronal networks. Nature 264: 705–12. 189195 10.1038/264705a0

[11] Paus T (2005) Mapping brain maturation and cognitive development during adolescence. Trends Cogn Sci 9: 60–68. 15668098 10.1016/j.tics.2004.12.008

[12] Steinberg LA (2008) A social neuroscience perspective on adolescent risk-taking. Dev Rev 28: 78–106. 18509515 10.1016/j.dr.2007.08.002 PMC2396566

[13] Chugani HT, Phelps ME, Mazziotta JC (1987) Positron emission tomography study of human brain functional development. Ann Neurol 22: 487–97. 3501693 10.1002/ana.410220408

[14] Jacobs B, Chugani HT, Allada V, Chen S, Phelps ME, et al (1995) Developmental changes in brain metabolism in sedated rhesus macaques and vervet monkeys revealed by positron emission tomography. Cereb Cortex 5: 222–33. 7613078 10.1093/cercor/5.3.222

[15] Harris LW, Lockstone HE, Khaitovich P, Weickert CS, Webster MJ, et al (2009) Gene expression in the prefrontal cortex during adolescence: implications for the onset of schizophrenia. BMC Med Genomics 2: 28. 19457239 10.1186/1755-8794-2-28 PMC2694209

[16] Somel M, Liu X, Tang L, Yan Z, Hu H, et al (2011) MicroRNA-driven developmental remodeling in the brain distinguishes humans from other primates. PLoS Biol (9) e1001214. 10.1371/journal.pbio.1001214 PMC3232219 22162950

[17] Whitford TJ, Rennie CJ, Grieve SM, Clark CR, Gordon E, et al (2007) Brain maturation in adolescence: concurrent changes in neuroanatomy and neurophysiology. Hum Brain Mapp 28: 228–37. 16767769 10.1002/hbm.20273 PMC6871488

[18] Feinberg I, Campbell IG (2010) Sleep EEG changes during adolescence: an index of a fundamental brain reorganization. Brain Cogn 72: 56–65. 19883968 10.1016/j.bandc.2009.09.008

[19] Casey BJ, Giedd JN, Thomas KM (2000) Structural and functional brain development and its relation to cognitive development. Biol Psychol 54: 241–257. 11035225 10.1016/s0301-0511(00)00058-2

[20] Casey BJ, Tottenham N, Liston C, Durston S (2005) Imaging the developing brain: what have we learned about cognitive development? Trends Cogn Sci 9: 104–110. 15737818 10.1016/j.tics.2005.01.011

[21] Blakemore SJ, Choudhury S (2006) Brain development during puberty: state of the science. Dev Sci 9: 11–14. 16445389 10.1111/j.1467-7687.2005.00456.x

[22] Spear LP (2000) The adolescent brain and age-related behavioral manifestations. Neurosci Biobehav Rev 24: 417–63. 10817843 10.1016/s0149-7634(00)00014-2

[23] Darmani NA, Shaddy J, Gerdes CF (1996) Differential ontogenesis of three DOI-induced behaviors in mice. Physiology and Behavior 60: 1495–500. 8946497 10.1016/s0031-9384(96)00323-x

[24] Adriani W, Chiarotti F, Laviola G (1998) Elevated novelty seeking and peculiar d-amphetamine sensitization in periadolescent mice compared with adult mice. Behav Neurosci 112: 1152–66. 9829793 10.1037//0735-7044.112.5.1152

[25] Laviola G, Macrì S, Morley-Fletcher S, Adriani W (2003) Risk-taking behavior in adolescent mice: psychobiological determinants and early epigenetic influence. Neurosci Biobehav Rev 27: 19–31. 12732220 10.1016/s0149-7634(03)00006-x

[26] Spear LP, Varlinskaya EI (2010) Sensitivity to ethanol and other hedonic stimuli in an animal model of adolescence: implications for prevention science? Dev Psychobiol 52: 236–43. 20222058 10.1002/dev.20457 PMC3045082

[27] Hascoet M, Colombel MC, Bourin M (1999) Influence of age on behavioural response in the light/dark paradigm. Physiology and Behavior 66: 567–70. 10386898 10.1016/s0031-9384(98)00333-3

[28] Macri S, Adriani W, Chiarotti F, Laviola G (2002) Risk taking during exploration of a plus-maze is greater in adolescent than in juvenile or adult mice. Anim Behav 64: 541–6.

[29] Terranova ML, Laviola G, de Acetis L, Alleva E (1998) A description of the ontogeny of mouse agonistic behavior. Journal of Comparative Psychology 112: 3–12. 9528111 10.1037/0735-7036.112.1.3

[30] Dyson SE, Jones DG (1980) Quantitation of terminal parameters and their inter-relationships in maturing central synapses: a perspective for experimental studies. Brain Res 183: 43–59. 7357409 10.1016/0006-8993(80)90118-3

[31] Adams I, Jones DG (1982) Quantitative ultrastructural changes in rat cortical synapses during early-, mid- and late-adulthood. Brain Res 239: 349–63. 7093695 10.1016/0006-8993(82)90514-5

[32] Markus EJ, Petit TL (1987) Neocortical synaptogenesis, aging, and behavior: lifespan development in the motor-sensory system of the rat. Exp Neurol 96: 262–78. 3569455 10.1016/0014-4886(87)90045-8

[33] Van Eden CG, Uylings HB (1985) Postnatal volumetric development of the prefrontal cortex in the rat. J Comp Neurol 241: 268–74. 4086657 10.1002/cne.902410303

[34] Somel M, Franz H, Yan Z, Lorenc A, Guo S, et al (2009) Transcriptional neoteny in the human brain. Proc Natl Acad Sci USA 106: 5743–5748. 19307592 10.1073/pnas.0900544106 PMC2659716

[35] Tseng KY, O'Donnell P (2007) Dopamine modulation of prefrontal cortical interneurons changes during adolescence. Cereb Cortex 17: 1235–40. 16818475 10.1093/cercor/bhl034 PMC2204087

[36] Heng L, Beverley JA, Steiner H, Tseng KY (2011) Differential developmental trajectories for CB1 cannabinoid receptor expression in limbic/associative and sensorimotor cortical areas. Synapse 65: 278–86. 20687106 10.1002/syn.20844 PMC2978763

[37] Engel AK, Fries P, Singer W (2001) Dynamic predictions: oscillations and synchrony in top-down processing. Nat Rev Neurosci 2: 704–716. 11584308 10.1038/35094565

[38] Varela F, Lachaux JP, Rodriguez E, Martinerie J (2001) The brainweb: phase synchronization and large-scale integration. Nat Rev Neurosci 2: 229–239. 11283746 10.1038/35067550

[39] Uhlhaas PJ, Roux F, Rodriguez E, Rotarska-Jagiela A, Singer W (2010) Neural synchrony and the development of cortical networks. Trends Cogn Sci 14: 72–80. 20080054 10.1016/j.tics.2009.12.002

[40] Whittington MA, Traub RD, Jefferys JG (1995) Synchronized oscillations in interneuron networks driven by metabotropic glutamate receptor activation. Nature 373: 612–615. 7854418 10.1038/373612a0

[41] Galiñanes GL, Taravini IR, Murer MG (2009) Dopamine-dependent periadolescent maturation of corticostriatal functional connectivity in mouse. J Neurosci 29: 2496–2509. 19244524 10.1523/JNEUROSCI.4421-08.2009 PMC2742915

[42] Kasanetz F, Riquelme LA, Murer MG (2002) Disruption of the two-state membrane potential of striatal neurones during cortical desynchronisation in anaesthetised rats. J Physiol 543: 577–589. 12205191 10.1113/jphysiol.2002.0024786 PMC2290508

[43] Gervasoni D, Lin SC, Ribeiro S, Soares ES, Pantoja J, et al (2004) Global forebrain dynamics predict rat behavioral states and their transitions. J Neurosci 24: 11137–11147. 15590930 10.1523/JNEUROSCI.3524-04.2004 PMC6730270

[44] Uylings HB, van Eden CG (1990) Qualitative and quantitative comparison of the prefrontal cortex in rat and in primates, including humans. Prog Brain Res 85: 31–62. 2094901 10.1016/s0079-6123(08)62675-8

[45] Uylings HB, Groenewegen HJ, Kolb B (2003) Do rats have a prefrontal cortex? Behav Brain Res 146: 3–17. 14643455 10.1016/j.bbr.2003.09.028

[46] Van De Werd HJ, Rajkowska G, Evers P, Uylings HB (2010) Cytoarchitectonic and chemoarchitectonic characterization of the prefrontal cortical areas in the mouse. Brain Struct Funct 214: 339–53. 20221886 10.1007/s00429-010-0247-z PMC2862954

[47] Seamans JK, Lapish CC, Durstewitz D (2008) Comparing the prefrontal cortex of rats and primates: insights from electrophysiology. Neurotox Res 14: 249–62. 19073430 10.1007/BF03033814

[48] Zold CL, Larramendy C, Riquelme LA, Murer MG (2007) Distinct changes in evoked and resting globus pallidus activity in early and late Parkinson's disease experimental models. Eur J Neurosci 26: 1267–1279. 17767504 10.1111/j.1460-9568.2007.05754.x

[49] Quiroga RQ, Nadasdy Z, Ben-Shaul Y (2004) Unsupervised spike detection and sorting with wavelets and superparamagnetic clustering. Neural Comput 16: 1661–1687. 15228749 10.1162/089976604774201631

[50] Bartho P, Hirase H, Monconduit L, Zugaro M, Harris KD, et al (2004) Characterization of neocortical principal cells and interneurons by network interactions and extracellular features. J Neurophysiol 92: 600–608. 15056678 10.1152/jn.01170.2003

[51] Fisher NI (1996) Statistical Analysis of Circular Data. Cambridge University Press.

[52] Cheetham CE, Fox K (2010) Presynaptic development at L4 to l2/3 excitatory synapses follows different time courses in visual and somatosensory cortex. J Neurosci 30: 12566–12571. 20861362 10.1523/JNEUROSCI.2544-10.2010 PMC2962420

[53] Kremer Y, Leger JF, Goodman D, Brette R, Bourdieu L (2011) Late emergence of the vibrissa direction selectivity map in the rat barrel cortex. J Neurosci 31: 10689–10700. 21775612 10.1523/JNEUROSCI.6541-10.2011 PMC6622639

[54] Clement EA, Richard A, Thwaites M, Ailon J, Peters S, et al (2008) Cyclic and sleep-like spontaneous alternations of brain state under urethane anaesthesia. PLoS One 3: e2004. 18414674 10.1371/journal.pone.0002004 PMC2289875

[55] Steriade M, Timofeev I, Grenier F (2001) Natural waking and sleep states: a view from inside neocortical neurons. J Neurophysiol 85: 1969–85. 11353014 10.1152/jn.2001.85.5.1969

[56] Destexhe A, Hughes SW, Rudolph M, Crunelli V (2007) Are corticothalamic 'up' states fragments of wakefulness? Trends Neurosci 30: 334–42. 17481741 10.1016/j.tins.2007.04.006 PMC3005711

[57] Raichle ME, Mintun MA (2006) Brain work and brain imaging. Annu Rev Neurosci 29: 449–76. 16776593 10.1146/annurev.neuro.29.051605.112819

[58] Contreras D, Destexhe A, Steriade M (1997) Spindle oscillations during cortical spreading depression in naturally sleeping cats. Neuroscience 77: 933–6. 9130774 10.1016/s0306-4522(96)00573-8

[59] Vincent JL, Patel GH, Fox MD, Snyder AZ, Baker JT, et al (2007) Intrinsic functional architecture in the anaesthetized monkey brain. Nature 447: 83–6. 17476267 10.1038/nature05758

[60] Buzsaki G, Draguhn A (2004) Neuronal oscillations in cortical networks. Science 304: 1926–1929. 15218136 10.1126/science.1099745

[61] Sowell ER, Thompson PM, Tessner KD, Toga AW (2001) Mapping continued brain growth and gray matter density reduction in dorsal frontal cortex: Inverse relationships during postadolescent brain maturation. J Neurosci 21: 8819–29. 11698594 10.1523/JNEUROSCI.21-22-08819.2001 PMC6762261

[62] Lebel C, Beaulieu C (2011) Longitudinal development of human brain wiring continues from childhood into adulthood. J Neurosci 31: 10937–47. 21795544 10.1523/JNEUROSCI.5302-10.2011 PMC6623097

[63] Sirota A, Montgomery S, Fujisawa S, Isomura Y, Zugaro M, et al (2008) Entrainment of neocortical neurons and gamma oscillations by the hippocampal theta rhythm. Neuron 60: 683–697. 19038224 10.1016/j.neuron.2008.09.014 PMC2640228

[64] Erickson SL, Lewis DA (2002) Postnatal development of parvalbumin- and GABA transporter-immunoreactive axon terminals in monkey prefrontal cortex. J Comp Neurol 448: 186–202. 12012429 10.1002/cne.10249

[65] Fries P (2009) Neuronal gamma-band synchronization as a fundamental process in cortical computation. Annu. Rev Neurosci 32: 209–224. 10.1146/annurev.neuro.051508.135603 19400723

[66] Tiitinen H, Sinkkonen J, Reinikainen K, Alho K, Lavikainen J, et al (1993) Selective attention enhances the auditory 40-Hz transient response in humans. Nature 364: 59–60. 8316297 10.1038/364059a0

[67] Herrmann CS, Munk MH, Engel AK (2004) Cognitive functions of gamma-band activity: memory match and utilization. Trends Cogn Sci 8: 347–355. 15335461 10.1016/j.tics.2004.06.006

[68] Gandal MJ, Edgar JC, Ehrlichman RS, Mehta M, Roberts TP, et al (2010) Validating gamma oscillations and delayed auditory responses as translational biomarkers of autism. Biol Psychiatry 68: 1100–1106. 21130222 10.1016/j.biopsych.2010.09.031 PMC5070466

[69] Spencer KM, Nestor PG, Perlmutter R, Niznikiewicz MA, Klump MC, et al (2004) Neural synchrony indexes disordered perception and cognition in schizophrenia. Proc Natl Acad Sci USA 101: 17288–17293. 15546988 10.1073/pnas.0406074101 PMC535363

[70] Cho RY, Konecky RO, Carter CS (2006) Impairments in frontal cortical gamma synchrony and cognitive control in schizophrenia. Proc Natl Acad Sci USA 103: 19878–19883. 17170134 10.1073/pnas.0609440103 PMC1750867

[71] Gonzalez-Burgos G, Lewis DA (2008) GABA neurons and the mechanisms of network oscillations: implications for understanding cortical dysfunction in schizophrenia. Schizophr Bull 34: 944–961. 18586694 10.1093/schbul/sbn070 PMC2518635

[72] Uhlhaas PJ, Singer W (2010) Abnormal neural oscillations and synchrony in schizophrenia. Nat Rev Neurosci 11: 100–113. 20087360 10.1038/nrn2774

[73] Nakazawa K, Zsiros V, Jiang Z, Nakao K, Kolata S, et al (2012) GABAergic interneuron origin of schizophrenia pathophysiology. Neuropharmacology 62: 1574–1583. 21277876 10.1016/j.neuropharm.2011.01.022 PMC3090452

[74] Hall MH, Taylor G, Sham P, Schulze K, Rijsdijk F, et al (2011) The early auditory gamma-band response is heritable and a putative endophenotype of schizophrenia. Schizophr Bull 37: 778–787. 19946013 10.1093/schbul/sbp134 PMC3122286

[75] Gallinat J, Winterer G, Herrmann CS, Senkowski D (2004) Reduced oscillatory gamma-band responses in unmedicated schizophrenic patients indicate impaired frontal network processing. Clin Neurophysiol 115: 1863–1874. 15261865 10.1016/j.clinph.2004.03.013

[76] Basar-Eroglu C, Brand A, Hildebrandt H, Karolina KK, Mathes B, et al (2007) Working memory related gamma oscillations in schizophrenia patients. Int J Psychophysiol 64: 39–45. 16962192 10.1016/j.ijpsycho.2006.07.007

[77] Spencer KM, Niznikiewicz MA, Shenton ME, McCarley RW (2008) Sensory-evoked gamma oscillations in chronic schizophrenia. Biol Psychiatry 63: 744–747. 18083143 10.1016/j.biopsych.2007.10.017 PMC2330275

[78] Norra C, Waberski TD, Kawohl W, Kunert HJ, Hock D, et al (2004) High-frequency somatosensory thalamocortical oscillations and psychopathology in schizophrenia. Neuropsychobiology 49: 71–80. 14981337 10.1159/000076413

[79] Flynn G, Alexander D, Harris A, Whitford T, Wong W, et al (2008) Increased absolute magnitude of gamma synchrony in first-episode psychosis. Schizophr Res 105: 262–271. 18603413 10.1016/j.schres.2008.05.029

[80] Whittington MA, Cunningham MO, LeBeau FE, Racca C, Traub RD (2011) Multiple origins of the cortical γ rhythm. Dev Neurobiol 71: 92–106. 21154913 10.1002/dneu.20814

[81] Akbarian S, Huang HS (2006) Molecular and cellular mechanisms of altered GAD1/GAD67 expression in schizophrenia and related disorders. Brain Res Rev. 52: 293–304. 10.1016/j.brainresrev.2006.04.001 16759710

[82] Lewis DA, Fish KN, Arion D, Gonzalez-Burgos G (2011) Perisomatic inhibition and cortical circuit dysfunction in schizophrenia. Curr Opin Neurobiol 21: 866–72. 21680173 10.1016/j.conb.2011.05.013 PMC3183273

[83] Light GA, Hsu JL, Hsieh MH, Meyer-Gomes K, Sprock J, et al (2006) Gamma band oscillations reveal neural network cortical coherence dysfunction in schizophrenia patients. Biol Psychiatry 60: 1231–1240. 16893524 10.1016/j.biopsych.2006.03.055

[84] Ferrarelli F, Massimini M, Peterson MJ, Riedner BA, Lazar M, et al (2008) Reduced evoked gamma oscillations in the frontal cortex in schizophrenia patients: a TMS/EEG study. Am J Psychiatry 165: 996–1005. 18483133 10.1176/appi.ajp.2008.07111733

[85] Kikuchi M, Hashimoto T, Nagasawa T, Hirosawa T, Minabe Y, et al (2011) Frontal areas contribute to reduced global coordination of resting-state gamma activities in drug-naive patients with schizophrenia. Schizophr Res 130: 187–194. 21696922 10.1016/j.schres.2011.06.003

[86] Liston C, Watts R, Tottenham N, Davidson MC, Niogi S, et al (2006) Frontostriatal microstructure modulates efficient recruitment of cognitive control. Cereb Cortex 16: 553–560. 16033925 10.1093/cercor/bhj003

[87] Galvan A, Hare TA, Parra CE, Penn J, Voss H, et al (2006) Earlier development of the accumbens relative to orbitofrontal cortex might underlie risk-taking behavior in adolescents. J Neurosci 26: 6885–6892. 16793895 10.1523/JNEUROSCI.1062-06.2006 PMC6673830

[88] Uhlhaas PJ, Roux F, Singer W, Haenschel C, Sireteanu R, et al (2009) The development of neural synchrony reflects late maturation and restructuring of functional networks in humans. Proc Natl Acad Sci USA 106: 9866–9871. 19478071 10.1073/pnas.0900390106 PMC2687997

[89] Ringli M, Huber R (2011) Developmental aspects of sleep slow waves: linking sleep, brain maturation and behavior. Prog Brain Res 193: 63–82. 21854956 10.1016/B978-0-444-53839-0.00005-3

