# Supplementary material for: Refinement of Neuronal Synchronization with Gamma Oscillations in the Medial Prefrontal Cortex after Adolescence
Source: PLoS One. 2013 Apr 30;8(4):e62978. doi: 10.1371/journal.pone.0062978 (PMC3639907; doi:10.1371/journal.pone.0062978)
Supplement: File S1 — This file contains figures relating to: Figure S1. Relative power spectra corresponding to LFPs recorded from the mPFC. Figure S2. Separation of putative cortical interneurons and pyramidal cells. Figure S3. Phase-locking analysis results for real and simulated cases. Figure S4. Representative traces. Figure S5. No significant correlation between anesthesia level and high-gamma entrainment in juvenile or adult mice. Figure S6. Lack of effect of anesthesia level on age dependent high-gamma neuronal entrainment. (PDF) [file pone.0062978.s001.pdf]

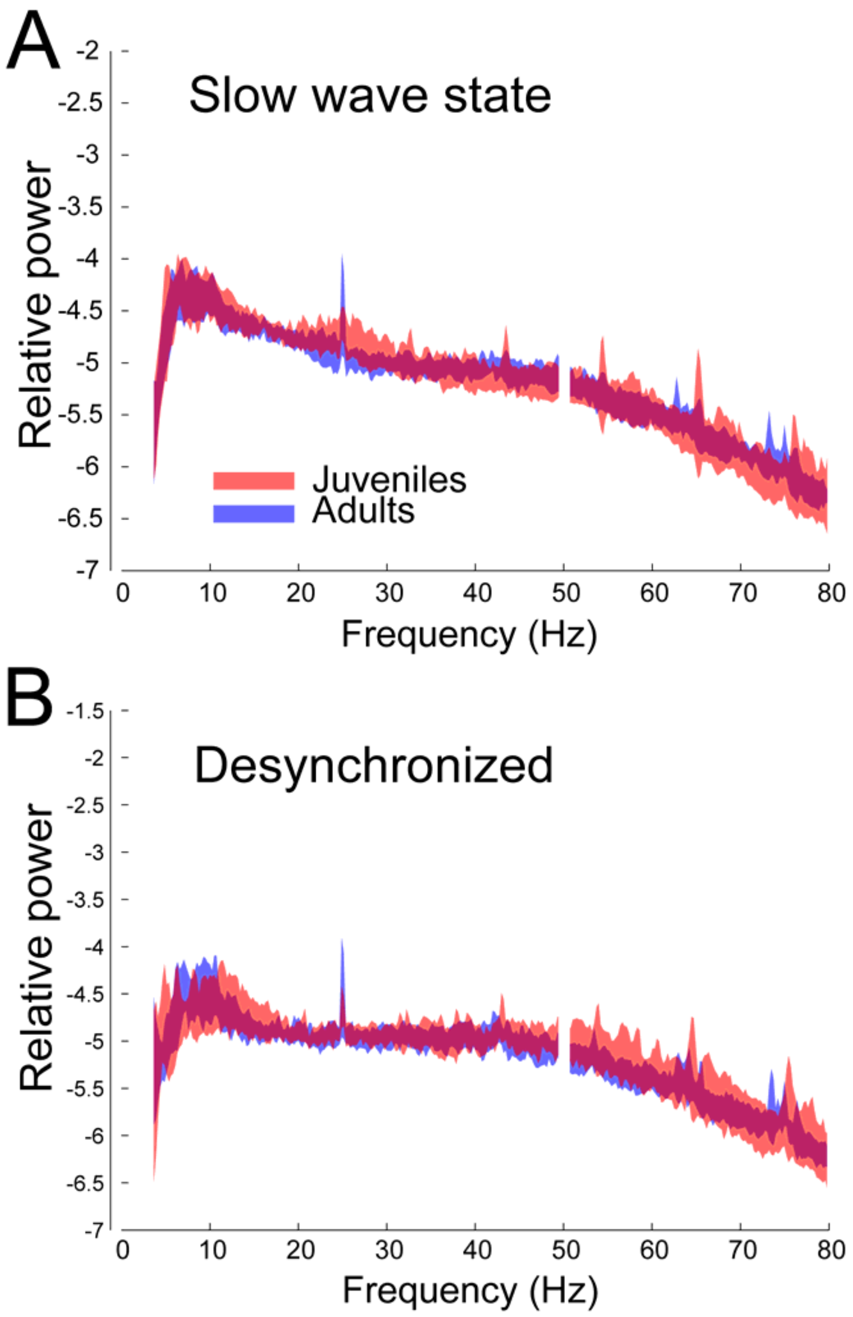

<u>Figure S1.</u>

Relative power spectra corresponding to LFPs recorded from the mPFC through the silicon multichannel electrode (95% confidence intervals), showing similar frequency composition in juveniles and adults in slow wave and desynchronized states.

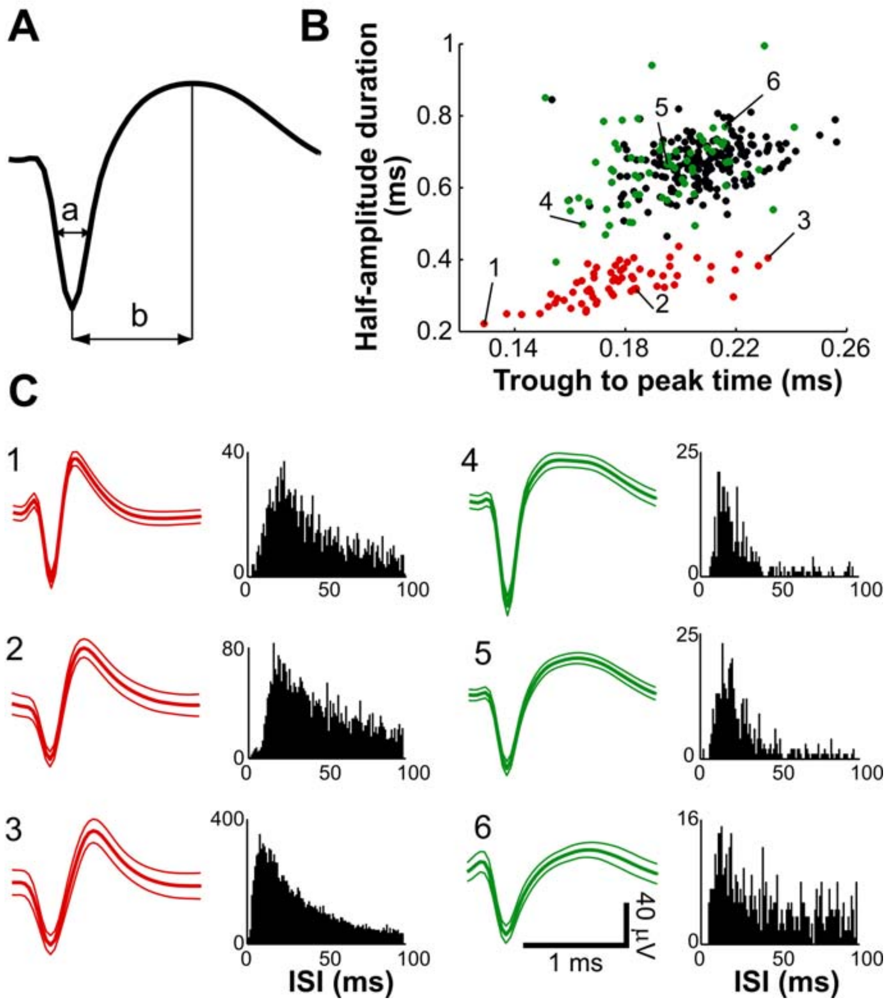

Figure S2.

Separation of putative cortical interneurons and pyramidal cells. A. Temporal parameters used in the clustering process from the average spike waveform of a single unit: (a) duration of the first spike component at half width and (b) trough to peak time. **B**. Neurons were clustered according to waveform parameters (a and b). All recorded units were plotted. Putative interneurons are shown in red, pyramidal neurons in green, and multiunit recordings in black. The numbers refer to the waveforms in (C). **C**. Individual examples extracted from (**B**), for each interneuron and pyramidal neuron, in addition to the waveform (mean +/- standard deviation), interspike interval distributions is also shown to evaluate the absence of spikes during refractory period.

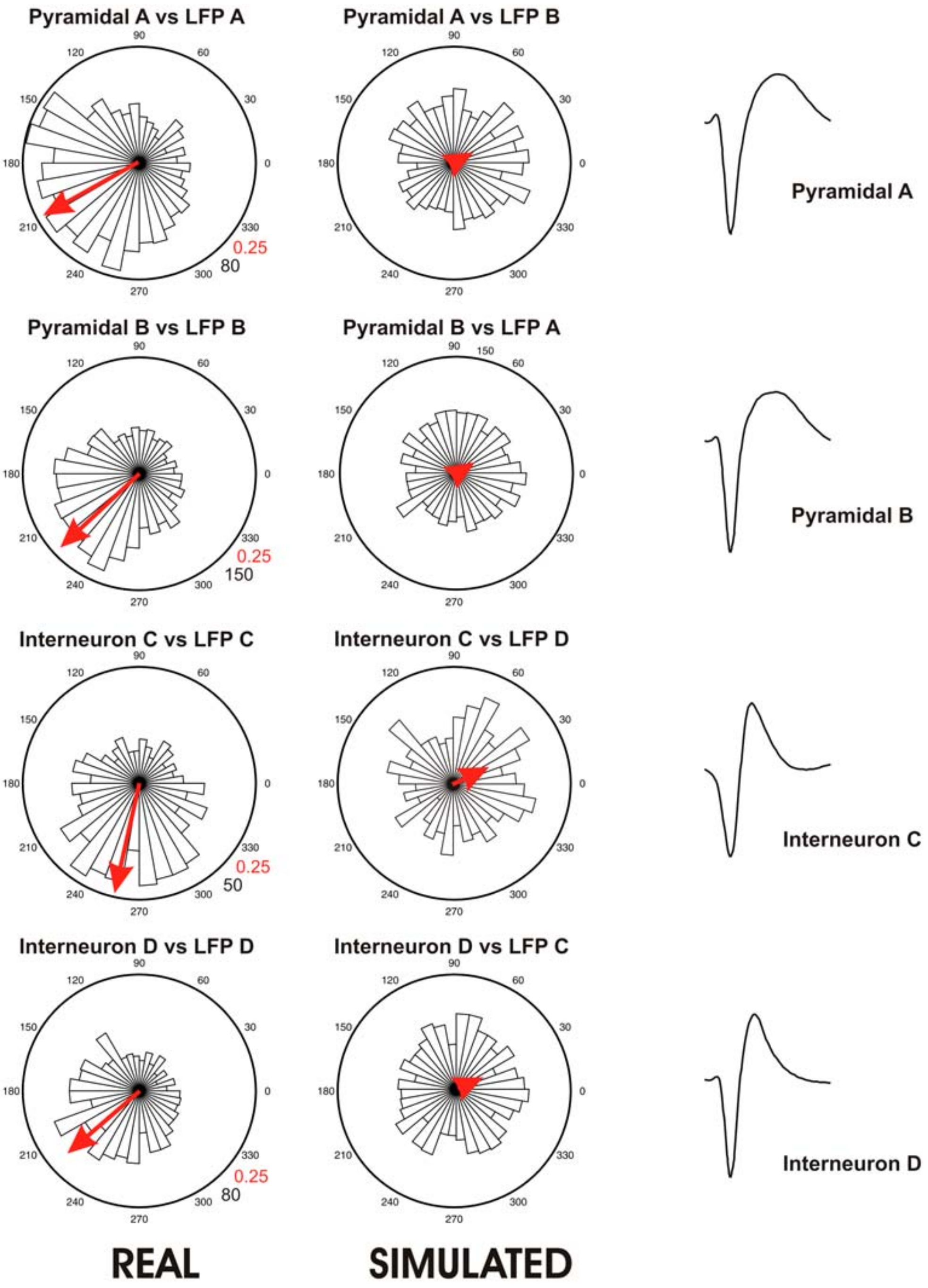

Figure S3.

Phase-locking analysis results for real and simulated cases. To evaluate contamination of LFP by spike waveforms pseudo-data were constructed using two pyramidal neurons (A and B) and two interneurons (C and D) from independent animals. Simulated signals were obtained by adding spikes waveforms extracted from neuron X, at the timestamps occurring in X, to the LFP signal recorded from Y (the inverse combination was also analyzed: waveforms and timestamps from Y were applied to LFP from X). Then, these simulated signals were processed identically to the real ones as described in materials and methods section. Spike-phase plots (bin size 10°) analysis shows a clear non-uniform distribution of spikes across phases for the high gamma band in the real cases (left column) and a uniform distribution for the same spikes in the simulated data (right column). The numbers at the lower right quadrant of the plots refer to the radial axes of frequency distribution and vector length. Pyramidal cells and interneurons were analyzed since spike waveforms differ between groups (average spike waveforms for each cell are presented on the right side) and LFP contamination after filtering may differentially affect each cell type.

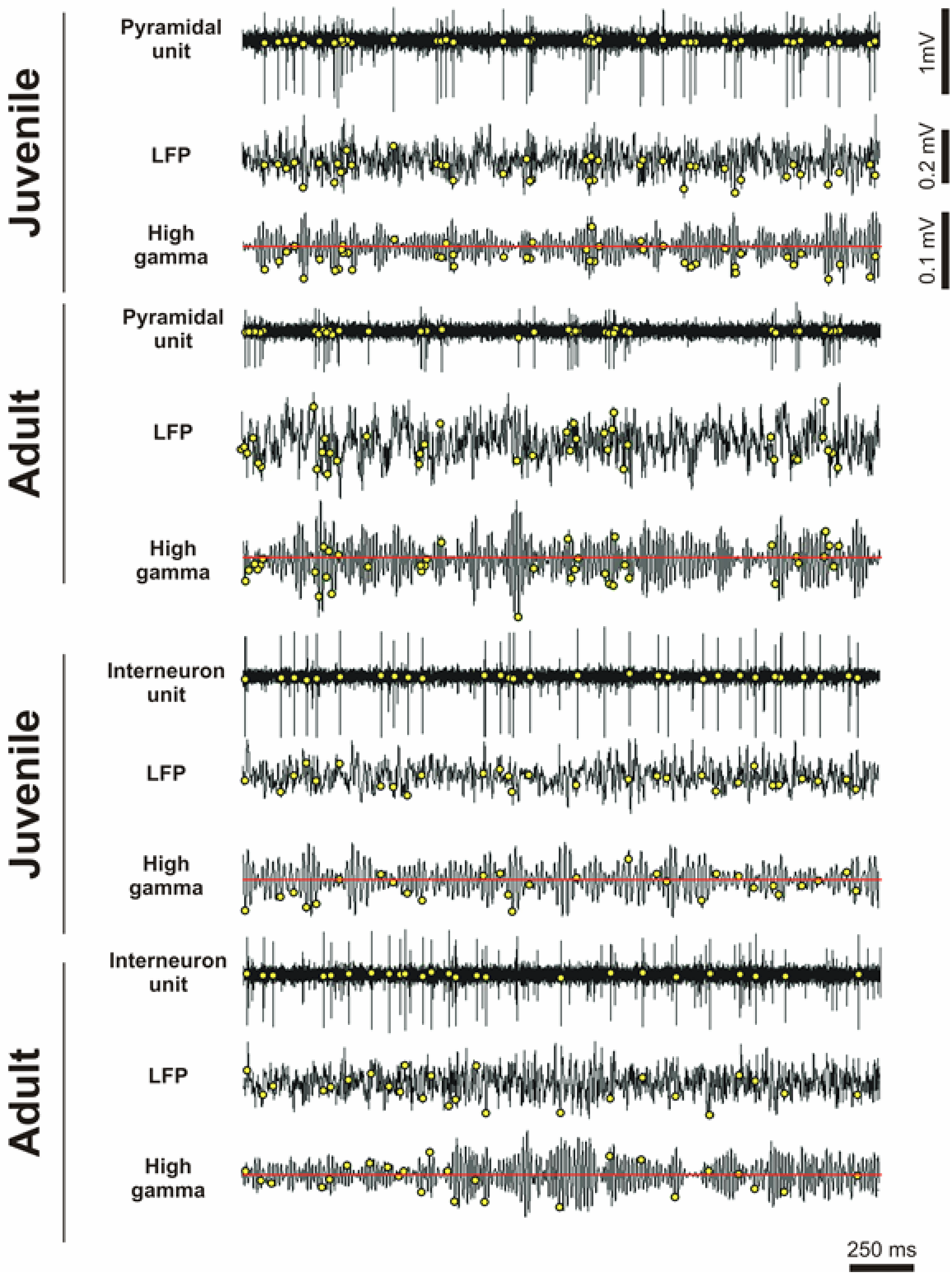

Figure S4.

Representative traces showing raw unitary activity for pyramidal cells and interneurons, local field potentials (LFP) and signals resulting from band-pass digital filtering the LFP for high gamma band (high gamma). The yellow dots mark the occurrence of spikes. Examples are provided from juvenile and adult mice.

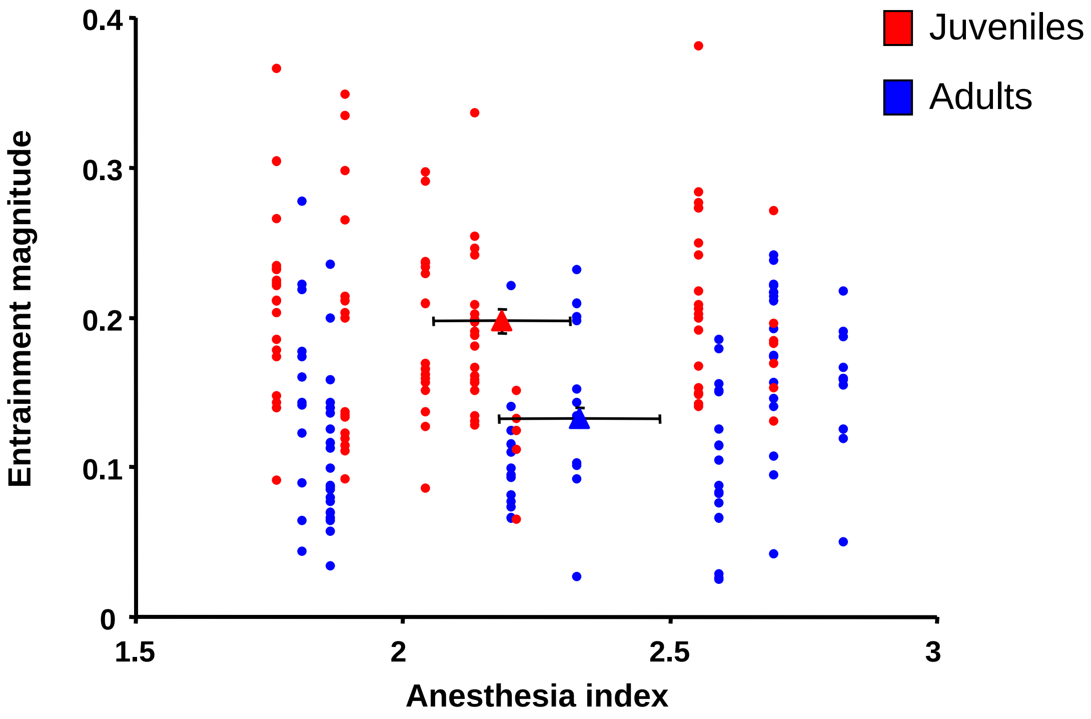

Figure S5.

No significant correlation between anesthesia level and high-gamma entrainment in juvenile or adult mice. Anesthesia depth was estimated by dividing the relative power of the slow wave (0.45-1.8 Hz band) of frontal cortex EEG by the relative power of theta (3.5-7 Hz band) from hippocampal EEG in one second intervals. An anesthesia index was then calculated for each animal by averaging the index across the 15-30 minutes recording session. Accordingly, the deeper the anesthesia, the higher the index. Each dot indicates the high-gamma entrainment magnitude of individual cells according to the corresponding anesthesia index (from 101 juvenile and 102 adults MUA recoding sites from 7 animals per group). Red and blue triangles represent mean ± sem values for juvenile and adult group. Pearson correlation indexes were calculated for each group: juvenile $r^2 = 0.033$; $t_{99} = 1.8$ $p = 0.1$ and adults $r^2 = 0.0007$; $t_{100} = 0.085$ $p = 0.9$.

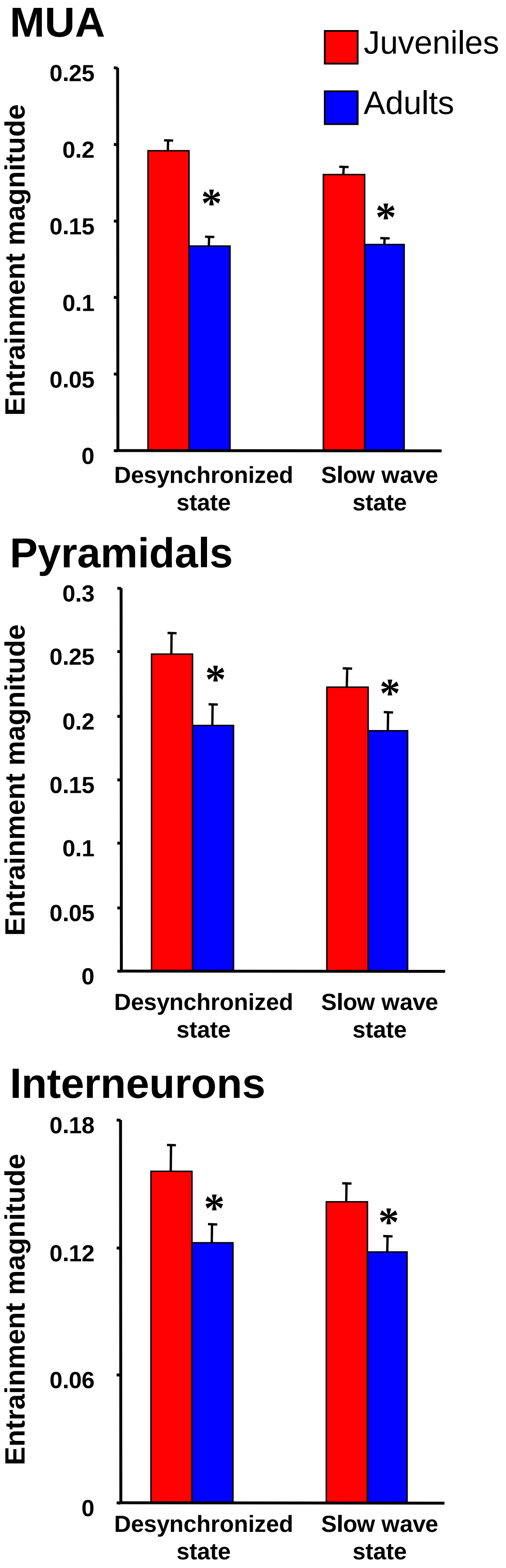

Figure S6.

Lack of effect of anesthesia level on age dependent high-gamma neuronal entrainment. High-gamma entrainment was calculated for multiunit recordings (MUA), pyramidal neurons and interneurons during two clearly different anesthetic depth levels, desynchronized and slow wave state. Anesthetic state was automatically determined by an algorithm described in materials and methods section in the main text of the manuscript. ANESTHESIA and AGE were used as factors in a two way ANOVA for each neuronal population. As presented in figure 4, a significant effect of AGE was observed for every recording type. However ANESTHESIA factor did not reach significance in any case. Interaction was also not significant, showing that neuronal entrainment to high-gamma oscillations depends on the developmental stage and not on depth of anesthesia.

* Results from two-way ANOVA

MUA

Factor:

AGE $F_{1,382}$ = 91.3 **p = 0.00001**

ANESTHESIA $F_{1,382}$ = 1.11 **p= 0.19**

AGE X ANESTHESIA $F_{1,382}$ = 1.98 **p= 0.16**

Pyramidals

Factor:

AGE $F_{1,130}$ = 7.15 **p = 0.008**

ANESTHESIA $F_{1,130}$ = 1.72 **p= 0.29**

AGE X ANESTHESIA $F_{1,130}$ = 0.65 **p= 0.42**

Interneurons

Factor: AGE $F_{1,125}$ = 7.01 **p = 0.009**

ANESTHESIA $F_{1,125}$ = 2.01 **p= 0.15**

AGE X ANESTHESIA $F_{1,125}$ = 0.96 **p= 0.32**
